# Fat-soluble vitamins: updated review of their role and orchestration in human nutrition throughout life cycle with sex differences

**DOI:** 10.1186/s12986-022-00696-y

**Published:** 2022-09-05

**Authors:** Rana A. Youness, Alyaa Dawoud, Omar ElTahtawy, Mohamed A. Farag

**Affiliations:** 1 Molecular Genetics Research Team (MGRT), Pharmaceutical Biology Department, Faculty of Pharmacy and Biotechnology, German University in Cairo, Cairo, Egypt; 2 Biology and Biochemistry Department, School of Life and Medical Sciences, University of Hertfordshire Hosted By Global Academic Foundation, Cairo, Egypt; 3 Biochemistry Department, Faculty of Pharmacy and Biotechnology, German University in Cairo, Cairo, Egypt; 4 Pharmacognosy Department, College of Pharmacy, Cairo University, Kasr El Aini St, Cairo, 11562 Egypt

**Keywords:** Fat-soluble vitamins, Daily requirements, Pregnancy, Lactation, Infants, Geriatrics, Sex

## Abstract

Age and Gender are vital determinants for the micronutrient demands of normal indviduals. Among these micronutrients are vitamins that are required in small amounts for optimum metabolism, homeostasis, and a healthy lifestyle, acting as coenzymes in several biochemical reactions. The majority of previous studies have examined such issues that relates to a specific vitamin or life stage, with the majority merely reporting the effect of either excess or deficiency. Vitamins are classified into water-soluble and fat-soluble components. The fat-soluble vitamins include vitamins (A, D, E, and K). Fat-soluble vitamins were found to have an indisputable role in an array of physiological processes such as immune regulation, vision, bone and mental health. Nonetheless, the fat-soluble vitamins are now considered a prophylactic measurement for a multitude of diseases such as autism, rickets disease, gestational diabetes, and asthma. Herein, in this review, a deep insight into the orchestration of the four different fat-soluble vitamins requirements is presented for the first time across the human life cycle beginning from fertility, pregnancy, adulthood, and senility with an extensive assessment ofthe interactions among them and their underlying mechanistic actions. The influence of sex for each vitamin is also presented at each life stage to highlight the different daily requirements and effects.

## Introduction

Despite its tiny molecular size, its physiological functions are indisputable. Vitamins are small organic molecules that are not synthesized endogenously in sufficient amounts which highlights their importance in our diet at all life stages, starting from neonatal to geriatric life [1]. Vitamins are organic essential micronutrients (needed at minute levels) that cannot be synthesized by vertebrates but are required to perform specific biological functions for normal growth and the maintenance of a human’s health [2].

The word “vitamin” was coined from two terms, vital and amine but due to the realization later on that, not all of them are amines the letter “e” was removed. The 13 known vitamins are classified according to their solubility in either water or fat which further affects their pharmacokinetic properties [3]. The fat-soluble vitamins (A, D, E, K) which are the main focus of this review are well-absorbed from the intestine in the presence of fats.

Historically, classical deficiencies of those vitamins were directly correlated with several pathological manifestations such as night blindness (due to Vitamin A deficiency), osteomalacia (due to Vitamin D deficiency), oxidative stress (due to Vitamin E deficiency), and hemorrhage (due to Vitamin K deficiency). However, over the past decade vitamins, A and D, in particular, have been associated with more complex disorders such as cancer and autoimmune diseases [4, 5]. In this review, a detailed description of each fat-soluble vitamin will be presented along with its role at every stage of human life and in the context of different-sex requirements.

## Vitamin A

Some vitamins are particularly made of one nutrient; this is not the case in vitamin A as it contains a broad group of related nutrients. The two main forms are retinoids (retinol, retinal, retinoic acid) and carotenes (α, β and γ); the latter refers to a pro-vitamin mainly found in plants, whereas the former is found in animals (non-vegetarian form) [6, 7].

### Vitamin A: metabolism, dietary sources, functions, and signaling pathways

**Fig. 1:**
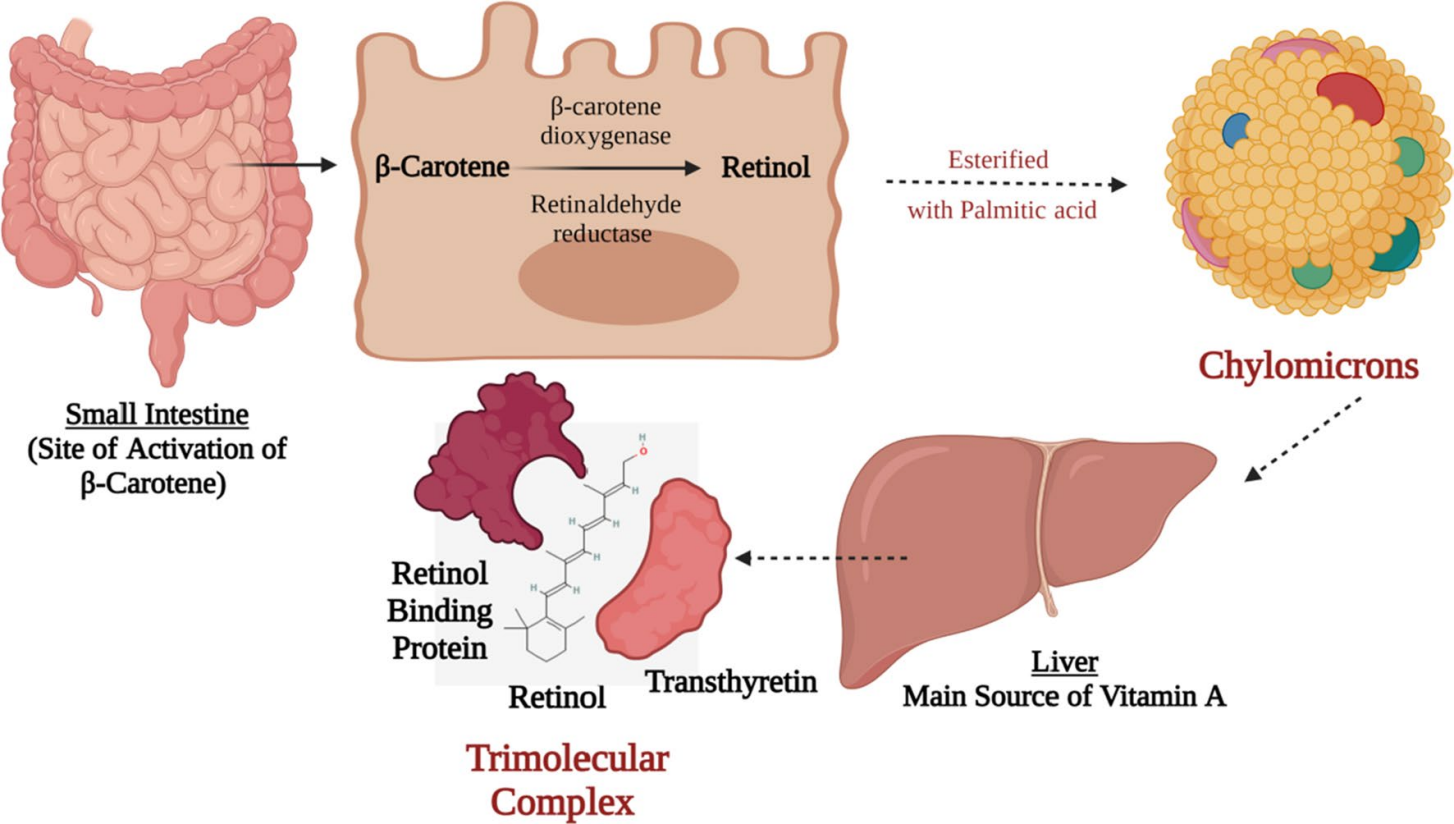
Vitamin A Biosynthesis and Activation. This figure represents a schematic presentation for the activation process of β-carotene to retinol in the small intestine following the absorption of the β-carotene from t;9he apical membrane and then the transportation of the activated retinol via the basolateral membrane directed towards the liver via chylomicrons. In the liver, it is processed in a trimolecular complex that is made up of active retinol, retinol-binding protein, and transthyretin protein. Such trimolecular complex prevents its glomerulus filtration via the kidneys

The main pro-vitamin is β-carotene and is transformed to its active form of vitamin A in the intestine where it is cleaved by β-carotene dioxygenase and by retinaldehyde reductase and finally reduced to retinol. Retinol is then esterified with palmitic acid into chylomicrons and with lipids to be delivered to the liver. The liver has around 90% of the vitamin A supply is secreted as retinol bound to retinol-binding protein (RBP), this complex combines with transthyretin (a protein) as presented in Fig. 1. This trimolecular complex acts to avoid vitamin A’s drainage by glomerulus filtration, and to halt the toxicity of retinol [8, 9].

**Fig. 2:**
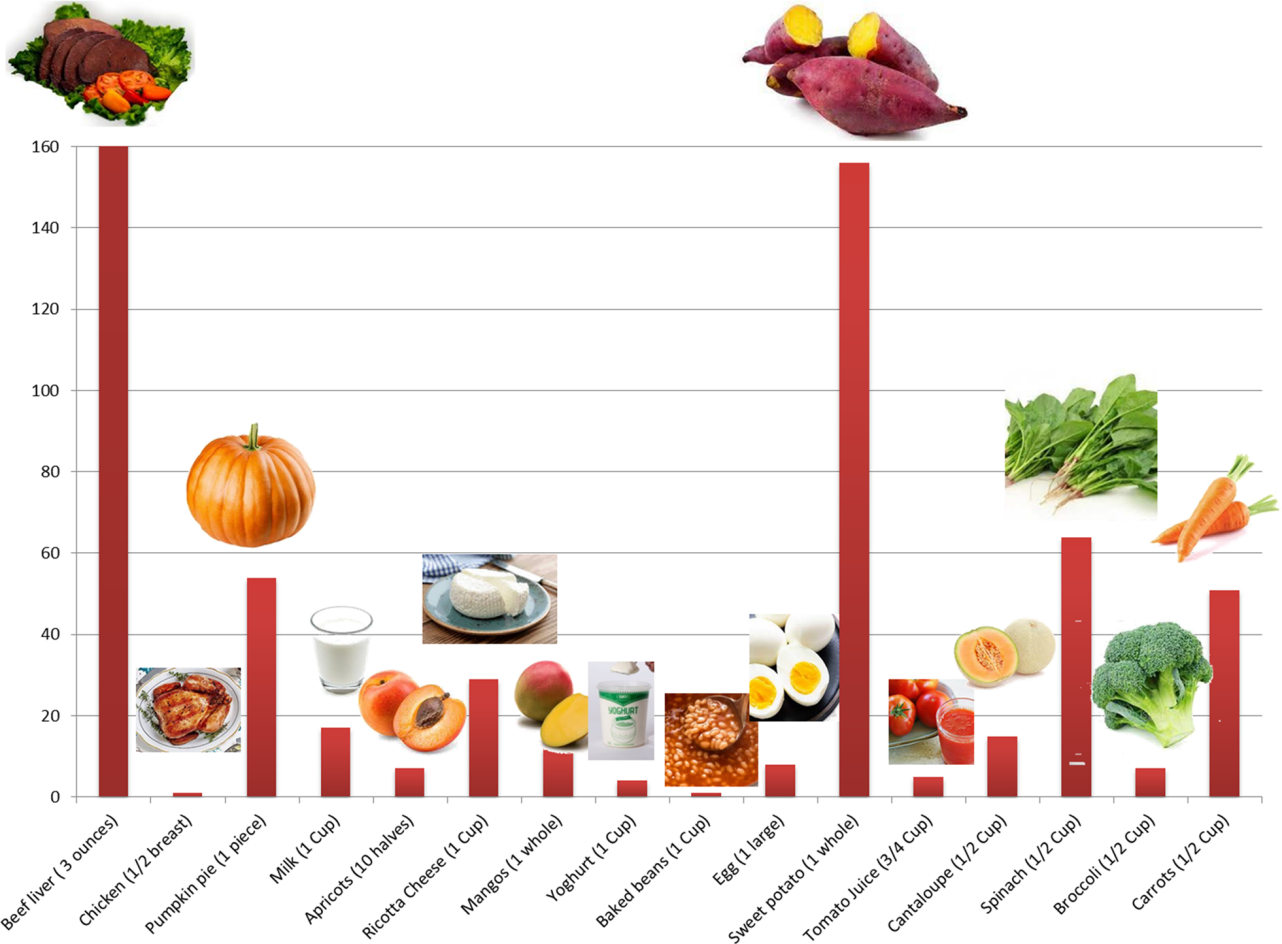
Vitamin A sources. A bar chart representing the sources of Vitamin A in daily food and its percentage daily value. Beef liver (3 oz) = 85.05gm; Chicken (½ breast) = 130gm; Pumpkin pie (1 piece) = 323gm; Milk (1 cup) = 240gm; Apricot (10 halves) = 105 gm; Ricotta Cheese (1 cup) = 220gm; Mangos (1 whole) = 336gm; Yoghurt (1cup) = 245gm; Baked beans (1 cup) = 172gm; Egg (1 large) = 50gm; Sweet potato (1 cup) = 133gm; Tomato juice (3/4 cup) = 169gm; Cantaloupe (½ cup) = 27gm; Spinach (½ cup) = 15gm; Broccoli (½ cup) = 35.5gm; Carrots (½ cup) = 64gm

It is also worth noting that vitamin A sources are diverse. It can be found in mammals as retinol and can be found in plants as carotenoids, the figure below (Fig. 2) shows different foods that are rich in vitamin A and their percentage daily value. On the biochemical and molecular levels, it has been reported that in a murine model that was used to investigate the effect of vitamin A on liver regeneration, vitamin A has been found to strongly affect the microbiota profile in treated animals and resulted in an enhancement of bile acid metabolism [10, 11]. On the other hand, gut microbiota (GM) have shown to be able to regulate vitamin A pharmacokinetic profile and immunomodulation functions [10, 12].

Vitamin A is essential in a wide spectrum of physiological activities such as clear retinal vision [13], skin health [14], immune system [15], reproduction [16], and embryonic development as previously reviewed in [16–19].

In early development stages throughout embryogenesis, vitamin A (retinol form) stimulates the activation of the transcription factor Hoxa1 which is important for segmentation and patterning of the hindbrain area as shown in Table 1 [20, 21]. During prenatal and postnatal life, it is well-known that either deficiency or excess of vitamin A might lead to birth defects (teratogenic effects). The signals initiated by the retinoids begin after the early phase of embryonic development (gastrulation) where retinoic acid is very important for organ development, including the heart, eyes, ears, lungs, limbs, visceral organs. Moreover, retinoid signaling is essential in the expression of many proteins of the extracellular matrix particularly collagen, laminin, and proteoglycans [22]. Such findings highlight the unquestionable role of Vitamin A in several cellular and molecular functions.

**Table 1:** Fat-soluble vitamins interactions with an array of signalling pathways at different cellular contexts

Vitamin	Cell types	Pathway	Reference
Vitamin A	Hindbrain area	Upregulates the transcription factor Hoxa1 → segmentation and patterning of the hindbrain area	[20, 21]
	Hepatic and colon cancer cells	Modulates the Expression of NKG2D Ligands → improving the targeting of tumor cells	[30]
	Extracellular matrix	Influences the expression of collagens, laminins, entactin, fibronectin, elastin and proteoglycans	[22]
	Hematopoietic stem cells	Regulates hematopoietic Stem Cell Dormancy → production of mature blood cells in bone marrow	[31]
	Immune cells	Contributes in immune cells maturation → improving immunity (anti-infective effect)	[23]
	Antigen presenting cells	Regulates antigen presenting cells → affecting immune regulation	[32]
	Spermatocytes	Inhibits spermatocytes apoptosis in early meiotic stages → survival of germ-cells and spermatogenesis	[33]
Vitamin D	Cell involved in inflammatory responses (neutrophils, monocytes, lymphocytes, and mast cells)	Downregulates COX-1 and COX-2 expression → affecting inflammatory response pathway	[34, 35]
	Ovarian cancer	Downregulates the telomerase enzyme → promoting cell apoptosis	[36]
	Ovarian cancer	Controls the expression of different regulatory molecules (HIF1a, p53, MYC, Ras, MAPK, BRCA1, and GADD45) → cell cycle arrest	[34]
	NSCLC	Interfering with HIF-1α/ VEGF axis → angiogenesis inhibition (antitumor effect)	[37]
	Spermatocytes	Increasing intracellular calcium concentration → mediating sperm motility	[38, 39]
	Male sex organs	Improves testosterone levels and erectile function	[40]
	GIT	Alteration in fecal microflora	[41, 42]
Vitamin E	Cells expressing Fatty acids translocase (FAT)/CD36 (platelets, mononuclear phagocytes, hepatocytes, adipocytes, myocytes, and some epithelia)	Modulates Fatty acids translocase (FAT)/CD36 scavenger receptor → Anti-oxidant activity	[43–46]
	T cell	Promotes IL-2 production → boostering T cell dividing capability (immunostimulant)	[47]
	Liver	Oxidized to α-TQ → lipotoxicity plasma biomarker in fatty liver subjects	[48, 49]
	GM in the intestine	Reduces the abundance of Lactobacillaceae and Bacteroides → affecting GM	[50]
Vitamin K	Endoplasmic reticulum of mammalian cells	Acts as a cofactor for the enzyme gamma glutamate carboxylase (GGCX) → regulates coagulation	[51]
	Vascular endothelial cells	Regulates calcification of vascular endothelial cells → increasing the risk of stroke and blood clots	[52]
	Brain cell membranes	Participates in sphingolipids biosynthesis → development of central nervous system (CNS) by participating in sphingolipids biosynthesis → they are essential component of the brain cell membranes	[53, 54]
	GM in the intestine	Affects the GM profile → decreasing the risk of colorectal cancer (anti-carcinogenic effect)	[55]

Furthermore, vitamin A contributes to immune system development [23]. Besides being well known as an anti-inflammatory vitamin [24], vitamin A participates in the maturation of immune cells and shows a potent anti-infective effects. It promotes T cells migration by inducing α4β7 expression level [25, 26], and increases B cell immunoglobulin production [27]. In a study performed on rabbits, in which their diet was enriched with carotenoids, serum levels of IgA, IgG, and IgM was elevated showing amelioration in their humoral immunity [27]. Concerning the anti-bacterial action of vitamin A, it has been noticed in a study on tuberculosis-model mice where administration of vitamin A derivatives has remarkably enhanced traditional anti-tuberculosis drugs’ efficacy [28]. Vitamin A exerts its bactericidal activity via inducing NPC2 expression level which in turn hinders cell wall biosynthesis in infective bacteria [29].

#### Preconception and pregnancy

Pregnancy is an important phase in the life of any woman during which the fate of her fetus is partially defined. During this period, specific nutritional needs are highly demanded to maintain good health for the mother and her fetus. For instance, vitamins demand/intake highly increases with different peaks at different trimesters based on the gradual fetal development [56].

Vitamin A is vital for the growth and maintenance of the fetus to provide a limited reserve in the fetus’s liver and for maternal tissue growth [57]. Vitamin A is not only essential for the morphological and functional development of the fetus, but is also vital for the ocular integrity and it exerts systemic effects on several fetal organs and the fetal skeleton [57]. Vitamin A has an indisputable role in ocular function, as it is involved in cell differentiation, maintenance of eye integrity, and prevention of xerophthalmia [58]. Yet, Vitamin A supplementation during pregnancy is a controversial issue and holds a lot of paradoxical facts.

In several developing countries, vitamin A deficiency is a public health problem being the primary cause of night blindness. On the contrary, in some developed countries, excessive Vitamin A intake during pregnancy has been directly linked with several teratogenic effects especially when administered in the 1st 60 days following conception. However, during the 3rd trimester of pregnancy vitamin A supplementation was found to decrease the incidence of broncho-pulmonary dysplasia in newborns [59].

Consequently, very strict vitamin A supplementation protocols for pregnant women have been conducted by the WHO and other health organizations [57]. In such context, WHO recommends supplementation of 3000 μg RAE/day or 7500 μg RAE/week during pregnancy in areas with the /high popularity of vitamin A deficiency (developing countries) to prevent night blindness in pregnant mothers an infant blindness, but not more than that because of the risk of teratogenicity (birth defects such as fontanelle) [60, 61]. However, the case in the developed countries is slightly different where it is recommended that monitoring of vitamin A intake whether from fortified foods or naturally should take place where it should not exceed 1500 μg RAE of vitamin A/day [60]. This underscores the importance of vitamin A as a vital vitamins for the growth and maintenance of the fetus to provide a limited reserve in the fetus’s liver and for maternal tissue growth [7]. Such discrepancy could be attributed to the different diets of people living in developed versus developing countries.

#### Lactation

“Mothers should absorb minerals and vitamins from their diets, to replace the amount lost in the milk produced to feed their infants”. This sentence has been widely used in folk medicine highlighting the fact that both the baby and the mother are prone to malnutrition during the lactation period if the intake of the vitamin was not closely monitored [7].

Vitamins are partially transferred to the fetus through placental transfer during the gestation period, but it is mainly transferred through the mother's milk during the neonatal period during lactation. Lactation is characterized by extensive morphological and metabolic changes in different tissues to guarantee a sufficient supply of blood and nutrients to the mammary gland for efficient milk production [56].

It is important to note that milk produced by the mother in the first days after birth (colostrum) is higher in proteins, vitamins A, B12, and K, and immunoglobulins and lower in fat content than mature milk 15 days after birth [62]. As previously mentioned retinol is released from the liver and incorporated in breast milk where it provides the infant with the required dosage to sustain visual, immune, and cognitive development. Vitamin A transfer through breast milk during the first 6 months of life is 60 times higher than the transfer occurring through the placenta during the 9 months of pregnancy. If the mother's vitamin A nutritional status is deficient, infants are susceptible to having vitamin A deficiency at the age of 6 months. Consequently, breastfeeding mothers and infants are considered at risk of vitamin A deficiency [63].

The concentration of vitamin A in human milk decreases throughout lactation; it is maximal in the colostrum and reaches a plateau in mature milk. In healthy mothers, vitamin A level varies from 5 to 7 μM in colostrum, 3 to 5 μM in transitional milk, and 1.4 to 2.6 μM in mature milk. The higher levels of retinol in colostrum allow tissue stores in the newborn to be rapidly replenished after limited placental transfer during gestation [56]. It is also noteworthy that low natural antibodies level could be a direct consequence of vitamin A deficiency in infants [64].

#### Infancy and childhood

Vitamin A supplementation during neonatal life is quite debatable, with one study suggesting that neonatal vitamin A supplementation was correlated with a 14% decrease in mortality by 6 months of age [65]. The WHO conducted three large studies targeting low-income countries in Africa and Asia to examine the accuracy of such hypothesis. This suggestion was disputed as it caused a bulging fontanelle in the newborn babies scoring a 0.1–1.2% and due to having this side effect they would need a long-term follow up to provide healthy development [65].

**Fig. 3:**
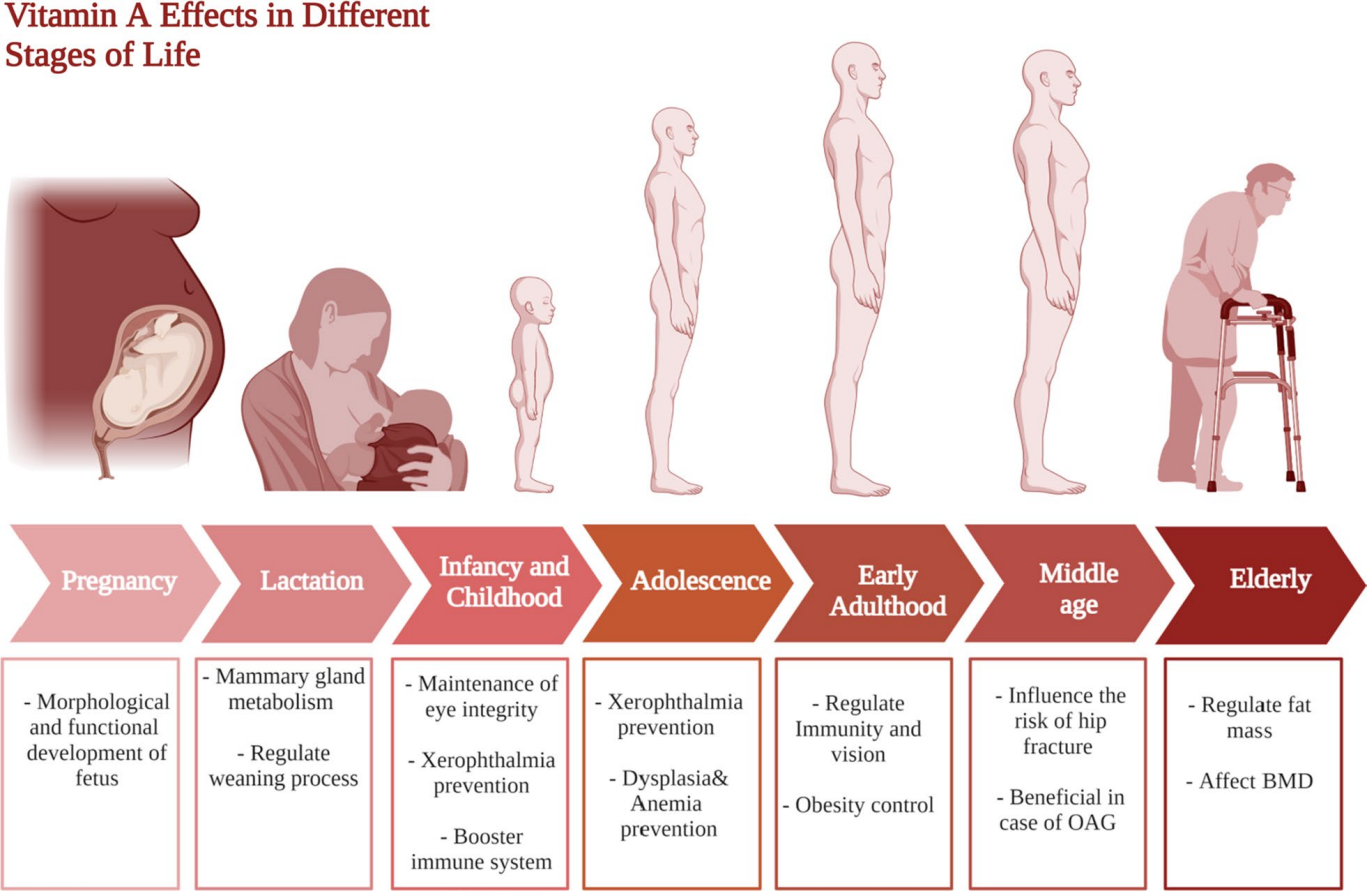
Vitamin A requirments in different stages of life. This figure represents a summary for vitamin A effects at different life stages starting form pregnancy and lactation till elderly

From 7–12 months, the average daily human milk consumption would be 650 ml and to provide 325 μg of vitamin A. Due to the increased risk of death from 6 months onwards in vitamin A-deficient populations, the requirements and recommendations of safe intake increased to 190 mg for 7 months of age and 400 mg for 12 months of age. It is generally recommended that children 1–3 years should take 300 mcg/day versus 400 mcg/day of vitamin A in 4–8 years, and 600 mcg/day in 9–13 years. Yet, due to the popularity of night blindness (1% or higher) in children at age 24–59 months, a high dosage of vitamin A supplementation is required and this is also applied in populations where infants, children, and mothers are infected with HIV. This has collectively been summarized in Fig. 3.

#### Adolescence

People between the age of 10 to 19 years old witness the most important physical and mental development which affect their life significantly [66]. Thus, adolescents’ health should be a great concern for health care to avoid future cognitive, developmental, health, and economic problems [67]. In addition, the significant increase in fast food consumption at this age in low- and middle-income countries, along with the unawareness of micronutrients importance, urges the need to pay more attention to applying healthy-diet programs [68, 69].

In a recent study that was performed on adolescent girls, results confirmed that deficiencies in vitamin A and E were common at this age likely attributed to the high growth requirements [70]. Vitamin A deficiency in adolescence is a major problem that exert a remarkable impact on the future life [71].

For vitamin A, it is involved in many critical physiological processes at this age with deficiency that could lead to visual rod cells damage in what is known as nyctalopia or night-blindness as shown in Fig. 3 [72]. Moreover, severe deficiency of vitamin A could lead to xerophthalmia which is irreversible blindness that is common in children and adolescents [72, 73].Furthermore, low vitamin A intake was linked to respiratory diseases recurrence and immune system compromising [74], in addition to dysplasia and anemia as shown in Fig. 3 [72]. Nonetheless, recent studies have revealed that vitamin A intake can reinstate hemoglobin levels if administered along iron supplements [75].

#### Adulthood

In middle age (between 40 and 60 years old), males and females are susceptible to physiological changes which are reflected in their metabolic profiles [76, 77]. It has been widely known that fat-soluble vitamin supplementation during adulthood serves as prophylactic treatment for numerous adverse health outcomes that could occur during middle age and elderly phases [78, 79].

As mentioned above, vitamin A has been long recognized for its role in immunity and vision warranting the importance of vitamin A supplementation [80]. However, studies highlight further effects of vitamin A in early adulthood. For example, a recent study found that a lower intake of vitamin A is associated with obesity in early adulthood [81]. In contrast, vitamin A might possess some alarming effects including toxicity to the mitochondria, furthermore decreased life quality and increased morbidity rates have been observed among vitamin A supplement users [82].

It is also worth mentioning that vitamin A supplementation has several unrelated roles during middle age. It may serve as a treatment or protective agent to several different ailments. Firstly, it has been shown to influence the risk of hip fractures [83]. It has been also linked to the decreased incidence of open-angle glaucoma (OAG) which is a fairly common health issue that many suffer from during middle age as summarized in Fig. 3 [84].

#### Elderly

Vitamin A requirements for adults aged 50 years old and higher have very little evidence; but toxicity can occur at lower doses. In addition, the influence of vitamins on senior population health is controversial which necessitates further investigations. A normal real dose of vitamin A (900 μg/day for women and 700 μg/day for men) is obtained by orally multivitamin/mineral labeled as vitamin A acetate that provides 750 μg [85]. A study on 200 women with mean age of 53.0 years old has revealed that normal levels of vitamin A are associated with overweight and obesity [86]. In addition, hypervitaminosis A above 3000 μg could lead to liver and kidney toxicities [85], whereas and unlike the previous studies, a recent study has reported that high vitamin A intake could show favorable outcomes on fracture risk [87].

#### Sex effect

Although the essentials of a healthy diet appear similar in both males and females. The essential requirements for micronutrients such as vitamins highly differ due to the great differences in their physiological and hormonal statuses. For instance, at the age of 40–60 years old, females experience menopause [88]. Waicek et al. performed a study to investigate changes in vitamin levels in menopausal women revealing that some vitamin levels are altered which necessitate following specific diet and vitamin supplements intake to avoid against future health complications [89].

On the molecular level, intracellular receptors of vitamin A, retinoic acid receptors (RARs) and retinoid X receptors (RXRs) have been found in different testis cell types at different life stages. These receptors affect gene expression by binding promotor regions [90, 91]. In a study on mice in which the gene encoding for RAR was knocked out, remarkable apoptosis of spermatocytes in early meiotic stages was monitored with germ-cell degeneration [33] suggesting that RAR plays a pivotal role in germ-cells survival and spermatogenesis [33].

In case of Vitamin A, recommended dietary allowance is 700 μg RAE/day in females versus 900 μg RAE/day for males for normal gene expression, immune function, and vision as collectively presented in Table 2.

**Table 2:** Recommended daily dose of fat-soluble vitamins by national institute of health (NIH)

	Pregnancy	Lactation	Infancy and childhood	Adolescence	Early adulthood	Middle age	Elderly
Vitamin A	11–19 years old: 750 µg RAE Older than 19 years old: 770 µg RAE	11–19 years old: 1200 µg RAE Older than 19 years old: 1300 µg RAE	0–6 months old: 400 µg RAE 7–12 months old: 500 µg RAE 1–3 years old: 300 µg RAE 4–8 years old: 400 µg RAE 9 years old: 600 µg RAE	10- 13 years old (M/F): 600 µg RAE 14 -18 years old (M): 900 µg RAE 14 -18 years old (F): 700 µg RAE 19 years old (M): 900 µg RAE 19 years old (F): 700 µg RAE	20–40 years old (M): 900 µg RAE 20–40 years old (F): 700 µg RAE	41–60 years old (M): 900 µg RAE 41–60 years old (F): 700 µg RAE	Older than 60 years old (M): 900 µg RAE Older than 60 years old (F): 700 µg RAE
Vitamin D	15 µg (600 IU)	15 µg (600 IU)	0–12 months old: 10 µg (400 IU) 1–9 years old: 15 µg (600 IU)	10–19 years old: 15 µg (600 IU)	20–40 years old: 15 µg (600 IU)	41–60 years old: 15 µg (600 IU)	61–70 years old: 20 µg (800 IU) Older than 70 years old: 20 µg (800 IU)
Vitamin E	15 mg	19 mg	0–6 months old: 4 mg 7–12 months old: 5 mg 1–3 years old: 6 mg 4–8 years old: 7 mg 9 years old: 11 mg	10–13 years old: 11 mg 14–19 years old: 15 mg	20–40 years old: 15 mg	41–60 years old: 15 mg	Older than 60 years old: 15 mg
Vitamin K	11–19 years old: 75 µg Older than 19 years old: 90 µg	11–19 years old: 75 µg Older than 19 years old: 90 µg	0–6 months old: 2 µg 7–12 months old: 2.5 µg 1–3 years old: 30 µg 4–8 years old: 55 µg 9 years old: 60 µg	10–13 years old: 60 µg 14 -18 years old: 75 µg 19 years old (M): 120 µg 19 years old (F): 90 µg	20–40 years old (M): 120 µg 20–40 years old (F): 90 µg	41–60 years old (M): 120 µg 41–60 years old (F): 90 µg	Older than 60 years old (M): 120 µg Older than 60 years old (F): 90 µg

(M): Male, (F): Female

## Vitamin D

Vitamin D is a vital micronutrient in all stages of life [92]. Despite being synthesized endogenously, 200–600 IU in different life stages is required daily as shown in Table 2 [93]. Meanwhile, vitamin D is extensively studied in literature owing to its numerous roles in human physiology [94]. Vitamin D is synthesized from cholesterol when the skin is exposed to the sun, it naturally exists in several forms including 7-dehydrocholesterol, vitamin D2, vitamin D3, 25-D, 1.25-D [95, 96].

### Vitamin D: metabolism, dietary sources, functions, and signaling pathways

Generally, vitamin D is metabolized through 3 main steps, which are 25-hydroxylation, 1 alpha-hydroxylation, and 24-hydroxylation [97]. These steps are all carried out by cytochrome P450 mixed-function oxidases (CYPs), which are present in the endoplasmic reticulum (ER) and mitochondria [97].

Vitamin D can be found in cod liver and fatty fish like salmon and mackerel [98]. Other fish, eggs, meat, dairy products, wild mushroom, and cultivated mushroom present good sources of vitamin D [98]. It plays a role in the reproduction process [99], bone health [100], prostate cancer [101], breast cancer [102, 103], depression and anxiety [104]. It is also worth noting that vitamin D plays a major role in calcium metabolism, regulation of immune cells functions, as well as hematopoietic cells differentiation and proliferation [105].

At the molecular level, vitamin D exerts its action through binding to vitamin D receptor (VDR) which is a transcription factor that regulates cells epigenetically. Ligand-activated VDR was found to affect the transcription of 1000 genes including genes involved in energy metabolism, calcium and bone homeostasis, as well as innate and adaptive immunity [106]. Moreover, vitamin D was found to downregulate COX-1 and COX-2 expression limiting their role in the inflammatory process as shown in Table 1 [34]. Investigation of cell-cycle controlling system has reported vitamin D participation in cell cycle arrest through controlling expression of different regulatory molecules such as HIF_1a_, p53, MYC, Ras, MAPK, BRCA1, and GADD_45_ [34]. Evidence has also revealed the potential role of vitamin D in promoting apoptosis by downregulating the telomerase enzyme [36]. Another antitumor effect for vitamin D was found via angiogenesis inhibition through interfering with HIF-1α/ VEGF axis [37].

Intriguingly, vitamin D was found to affect and get affected by GM, too [10]. Alteration in fecal microflora was reported upon treatment with vitamin D [41, 42]. Yet, GM has been reported to be capable of manipulating vitamin D activity either through upregulating VDR intestinal expression by producing short-chain fatty acid such as butyrate [107], or through competing it for VDR by lithocholic acid, a secondary bile acid [108].

### Vitamin D profile at different life stages

#### Preconception and pregnancy

Vitamin D supplementation during pregnancy has been shown to exert crucial effects on the fetus’s status in terms of skeletal health, immune system, as well as protecting the mother’s health [109, 110]. Furthermore, vitamin D deficiency is common among pregnant women and is associated with several adverse outcomes such as preeclampsia, gestational diabetes, preterm births, and low birth weights [111, 112].

Moreover, vitamin D plays a very significant immunoregulatory role in pregnancy. During pregnancy, the maternal immune system needs to tolerate the fetus. A group of the T lymphocytes, called regulatory T cells (Tregs) of potential immunosuppressive activity responsible for decreasing the destructive effect of the maternal immune system to her fetus [109]. It has been found that vitamin D increases the number of Tregs, in turn decreasing the chance of autoimmune diseases occurrence such as rheumatoid arthritis (RA), systemic lupus erythematosus (SLE), antiphospholipid syndrome (APS), Hashimoto Thyroiditis (HT), and multiple sclerosis (MS) [109].

It has been established that vitamin D alongside calcium plays an important role in skeletal health [113]. Expectedly, several recent reviews have concluded that vitamin D maternal supplementation is essential for healthy fetal bone growth through improving vitamin D and calcium levels in infants [110, 114]. Furthermore, low vitamin D levels in pregnant women are associated with an increased risk of fatal asthma, preeclampsia, gestational diabetes, preterm births, low birth weight wheeze, respiratory tract infections, and eczema [110, 111, 115–117].

No agreement has been reached regarding the healthy vitamin D dose per day for pregnant women. For instance, the Institute of Medicine set the healthy dose at 600 IU (international units) per day, while the Endocrine society sets it at 1500- IU per day, others have suggested ranges of 800–1200 IU and 400–800 IU per day for pregnant women [62, 110]. It is also noteworthy to mention that a recent review found conflicting conclusions regarding the main carrier of vitamin D, Vitamin D binding protein (VDBP) [118]. It was found that low levels of maternal serum VDBP are associated with several adverse pregnancy outcomes such as spontaneous miscarriage and pre-eclampsia, nevertheless increased VDBP levels in the cervicovaginal fluid were linked to an unexplained miscarriage [118].

#### Lactation

**Fig. 4:**
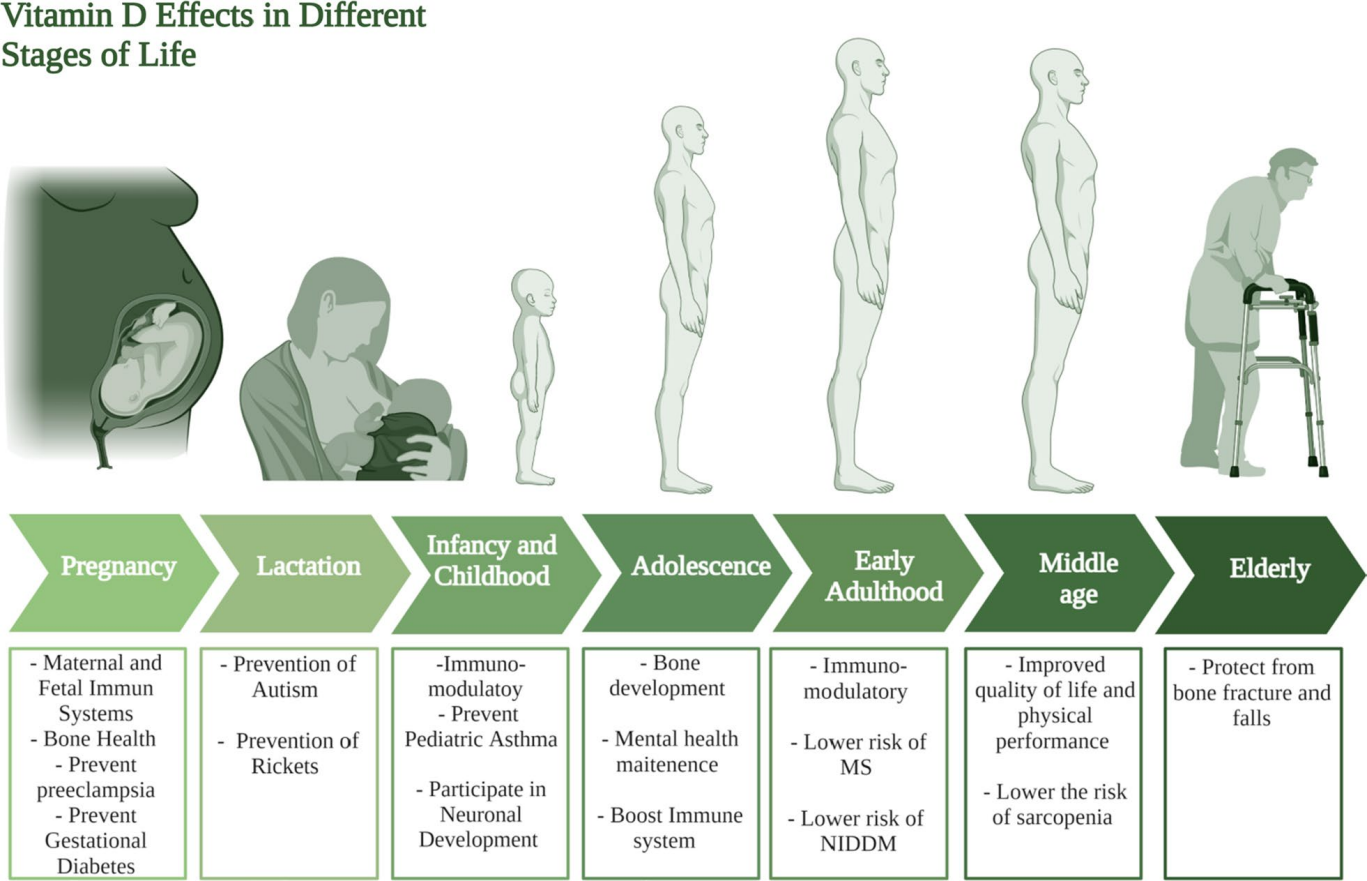
Vitamin D requirments at different stages of life. This figure represents a summary for vitamin D effects at different life stages starting form pregnancy and lactation till the elderly population

The role of vitamin D supplementation for lactating women can be viewed as a continuation of its role during pregnancy, the neonate needs vitamin D for the same reasons they needed for during pregnancy. However, one of the novel roles of vitamin D during lactation lies in the prevention and treatment of autism. Several studies point out the increased incidence of autism in vitamin D deficient children [119]. In such context, it is referred to vitamin D as a neuro-steroid that plays an important role in several process of brain development [119]. It was also found that children who are autistic have lower levels of 25(OH)D levels (the barometer of vitamin D level) at 3 months and age 8 compared to their non-autistic siblings [119]. Collectively, these studies show how vitamin D supplementation during lactation may significantly decrease the incidence of autism. Another important role of vitamin D in lactation is the prevention of rickets in infants [120]. Several reports found that vitamin D deficient neonates suffer from a higher risk of developing several disorders including rickets as summarized in Fig. 4 [120, 121].

Different sources suggest different amounts and methods of vitamin D supplementation during lactation [122, 123]. For example one study concluded that pregnant and lactating women should take (5000 IU/day) of vitamin D to prevent autism [119]. A randomized controlled trial found that supplying the mother with (6400 IU/day) of vitamin D per day satisfies her infant’s daily intake requirement [124]. Other reports suggest that infant’s supplementation of vitamin D through breastfeeding is not enough and will lead to vitamin D deficiency [123]. It is also noteworthy to mention that in the last 15 years, poison control reports have recorded 15,000 cases of vitamin D overdose, out of which no one died, showing that vitamin D is indeed safe [119].

#### Infancy and childhood

As an extrapolation of its high demand during the infancy stage, vitamin D is still of vital importance during the whole childhood period. Besides autism, Vitamin D has been found to play several roles in pediatric diseases, one of which is asthma. A recent review concluded that vitamin D supplementation helps in the prevention of the development of asthma [125]. Likewise a randomized, placebo-controlled clinical trial was conducted on school children who suffer from asthma, the trial showed that vitamin D supplementation as an adjunct therapy may help control asthma as summarized Fig. 4 [126].

Respiratory tract infections (RTIs) represent a great burden as they are the most common cause of morbidity among children under 5 years old [127]. Vitamin D level was found to be low in most infants and children who suffer from RTIs [127], and in accordance with other studies confirming that vitamin D supplementation may serve for prophylaxis against some RTIs [128, 129].

Another aspect in which vitamin D plays an important role is bone acquisition and development. Low levels of vitamin D are involved in the development of extra skeletal problems in childhood [130, 131].

Additionally, Vitamin D has been reported to be a vital modulator of neurodevelopment [132], with neurotransmission, oxidative stress, and neural cell proliferation as the most important mechanisms modulated by vitamin D [132]. Subsequently, its deficiency during early childhood negatively affects the developing brain, possibly leading to autism spectrum disorder (ASD) [132]. Furthermore, recent studies shed light onto the relationship between vitamin D deficiency and the development of several mental disorders in children [133]. Collectively, it can be concluded that vitamin D possesses an important preventative/ curative / modulatory role. Yet, regarding its dose, different institutions have opposing opinions about the correct dose of vitamin D that could be due to different geographical characteristics for the participants in each study [134, 135].

#### Adolescence

During adolescence, vitamin D is vital for bone development being considered as one of the most crucial micronutrient in this phase at which rapid bone development occurs [136]. The recommended dose for adolescents of vitamin D is 600 IU per day, however, the safety margin of vitamin D intake reaches up to 4,000 IU per day [137]. In addition, hypovitaminosis D in early life stages such as in adolescence was linked to mental disorders [133]. Vitamin D was also found to be associated with immune system wellbeing at this stage of life [138].

#### Adulthood

It has been reported that it is quite essential to keep adequate levels of vitamin D throughout all different stages of life as high serum 25-OHD levels in youth to adulthood [139]. In this stage of life, it has been repeatedly reported the immune-modulatory role of vitamin D, as it decreases excessive inflammation [140]. It is also associated with a decreased risk of developing type 2 diabetes in individuals in their adulthood period as summarized in Fig. 4 [141]. Noteworthy, low vitamin D levels in early life have been directly associated with a risk of multiple sclerosis later in life, however further studies are required to confirm these findings [142].

Vitamin D supplementation serves as a prophylactic treatment for several diseases common for middle-aged individuals. A clinical trial conducted by Manoy et al. [143] revealed that vitamin D2 supplementation (40,000 IU per week) for 6 months decreased oxidative protein damage, improved quality of life and physical performance in osteoarthritis patients. It has been recently reported that vitamin D plays a vital role in alleviating several sleep disorders and chronic pain conditions [144].

#### Elderly

With the remarkable pace by which life expectancy gains years [145], the demand of micronutrients increases parallel to the decrease in nutrients digestion and food intake from which elderly people suffer from [146]. Consequently, it’s not surprising that in a study investigating the status of different vitamins in elderly population and the effect of vitamin supplements on their health, it was found that 88% of subjects were deficient in vitamin D while 42% were deficient in vitamin K [147], and justifying the importance of regular vitamin intake in this age to avoid many complication that could result from vitamin deficiency or overdoses [147].

Vitamin D deficiency expose senior population to many risks including bone fracture upon falling [92]. A meta-analysis revealed that the risk of falling in senior population is reduced by 19% when vitamin D reaches 17.5–25 μg per day. Consequently, the risk of hip fracture declines [148]. Another clinical trial on institutionalized patients reported that 20 μg of vitamin D combined with 1200 mg calcium per day have resulted in lowering falls and fracture risk. However, lower doses of vitamin D and calcium, 10 μg and 1000 mg per day, respectively, have shown the same outcomes in people older than 65 years [149, 150].

#### Sex effect

It has been reported that males at younger age are more vulnerable to vitamin D deficiency than females [151]. Vitamin D level has also shown no relevance to all-types of cancer (excluding non-melanoma skin cancers) prevention in senior female population [152]. However, optimum concentration of vitamin D and calcium should be adjusted to avoid the risk of colorectal cancer development among males [153]. Nevertheless, vitamin D is believed to affect males’ fertility by affecting semen quality and sex steroids production [38]. Besides, VDR was found to mediate sperm motility via increasing intracellular calcium concentration [38, 39]. In this context, treatment of vitamin D deficient middle-aged male subjects with vitamin D for 12 month has been found to improve their testosterone levels and erectile function [40].

## Vitamin E

Vitamin E is present in different forms such as α-, β-, γ-, δ-, tocopherol and tocotrienols. However, α-tocopherol is considered as the most active form of vitamin E [154], mostly recognized for its antioxidant action. Vitamin E acts mainly as antioxidant to protect the cell from reactive oxygen and nitrogen species (ROS and RNS) along with other antioxidants (e.g., vitamin C, glutathione) and enzymes (e.g., peroxides, catalase) [154]. Vitamin E has also been found to regulate platelets aggregation by the following mechanism; where prostacyclin release is promoted via vitamin E in the endothelial cells, prostacyclin inhibits the aggregation of platelets [154]. Nevertheless, vitamin E was reported to be a suppressor for leukotrienes synthesis exerting an anti-inflammatory effect [85]. In addition, recent studies have shown that different forms of vitamin E have the ability to reduce the risk of cancer development [155].

### Vitamin E: metabolism, dietary sources, functions, and signaling pathways

Similar to other fat soluble vitamins, vitamin E is absorbed in chylomicron micelles from the intestines, then, released to the bloodstream by the action of lipoprotein lipase or it goes to the liver and released to the blood stream in form of very-low-density lipoprotein (VLDL) to be eventually stored in the adipose tissue [85]. Vitamin E catabolism usually begins with the rate limiting step in which ω-hydroxylation takes place as mediated via CYP4F2/3A4 enzymes [156]. The metabolites are then conjugated and excreted whether in feces or urine [156, 157].

Similar to other fat-soluble vitamins, vitamin E shows a cross interaction with GM [10]. A study by Choi et al. on mice confirmed differences between GM profiles of high dietary α–tocopherol group and the other which intakes lower dose [158]. Likewise, in a study on male subjects, intake of 400 IU of α–tocopherol form of vitamin E twice daily affected GM by reducing Lactobacillaceae and Bacteroides abundance [50]. Studies further revealed that GM could alter vitamin E lifespan in the intestine by activating its metabolism by CYP450-dependent pathway [159].

Vitamin E can be found in large quantities in legume seeds, almond, butter, tomato, and leafy vegetables (e.g., beet greens, collard greens, spinach) [85]. It also can be found in oils such as sunflower, safflower, soybean oil, and the germ oil of cereal grains [160, 161]. Concerning the daily requirements of vitamin E among both sexes and at different life stages, it has been summarized in Table 2 and explained below.

Anti-oxidant activity of vitamin E has been studied intensively and correlated mainly to CD36 scavenger receptor and Fatty acids translocase (FAT) modulation as shown in Table 1 [43–45]. Vitamin E was reported as immunostimulatn by promoting interleukin (IL)-2 production, boostering T cell dividing capability [47]. On the other hand, vitamin E oxidized metabolite α-tocopheryl quinone (α-TQ) has been reported to be an emerging plasma biomarker of lipotoxicity in subjects with fatty liver [48]. In a study on fatty liver patients, α-TQ was positively correlated with liver dysfunction and metabolic failure, which could help in early prediction of non-alcoholic fatty liver disease (NAFLD) and nonalcoholic steatohepatitis (NASH) progression [48, 49].

### Vitamin E profile at different stages of life

#### Preconception and pregnancy

Conflicting results have been presented regarding the role of vitamin E in pregnancy. One study suggests that it has antioxidant properties, hence combatting the oxidative stress in the body [162]. An increased level of oxidative stress may lead to pre-eclampsia and premature rupture of membranes (PROM) [162]. Another hypothesizes that vitamin E supplementation during pregnancy does not prevent pre-eclampsia or PROM [163]. Furthermore, a recent review that assessed several trials reached the conclusion that supplementation of any vitamins before or during pregnancy does not prevent the occurrence of miscarriages [164]. On another note, a recent study that included 1924 children found that lower maternal vitamin E supplementation during pregnancy was associated with an increased risk of children being diagnosed with asthma in their first 10 years [165]. To sum up, the role of vitamin E during pregnancy remains controversial, and further clinical investigations are needed to uncover it.

#### Lactation

In an observational study in which very low birth weights infants’ plasma retinol and alpha-tocopherol levels were measured, at different stages during lactation; vitamin E deficiency has been found to correlate with very low birth weights in new born infants [166]. Physiologically, the level of vitamin E in the milk tends to decrease with the progression of lactation, highlighting the importance of modifying and increasing the recommended dose of vitamin E supplementation [167]. Other factors to influence vitamin E content of the milk include mother’s age, the mother’s vitamin E supplementation, maternal obesity, smoking and the gestational age [167, 168]. Therefore, a customized supplementation of vitamin E should be prescribed to the lactating mother based on her status and medical conditions.

#### Infancy and childhood

**Fig. 5:**
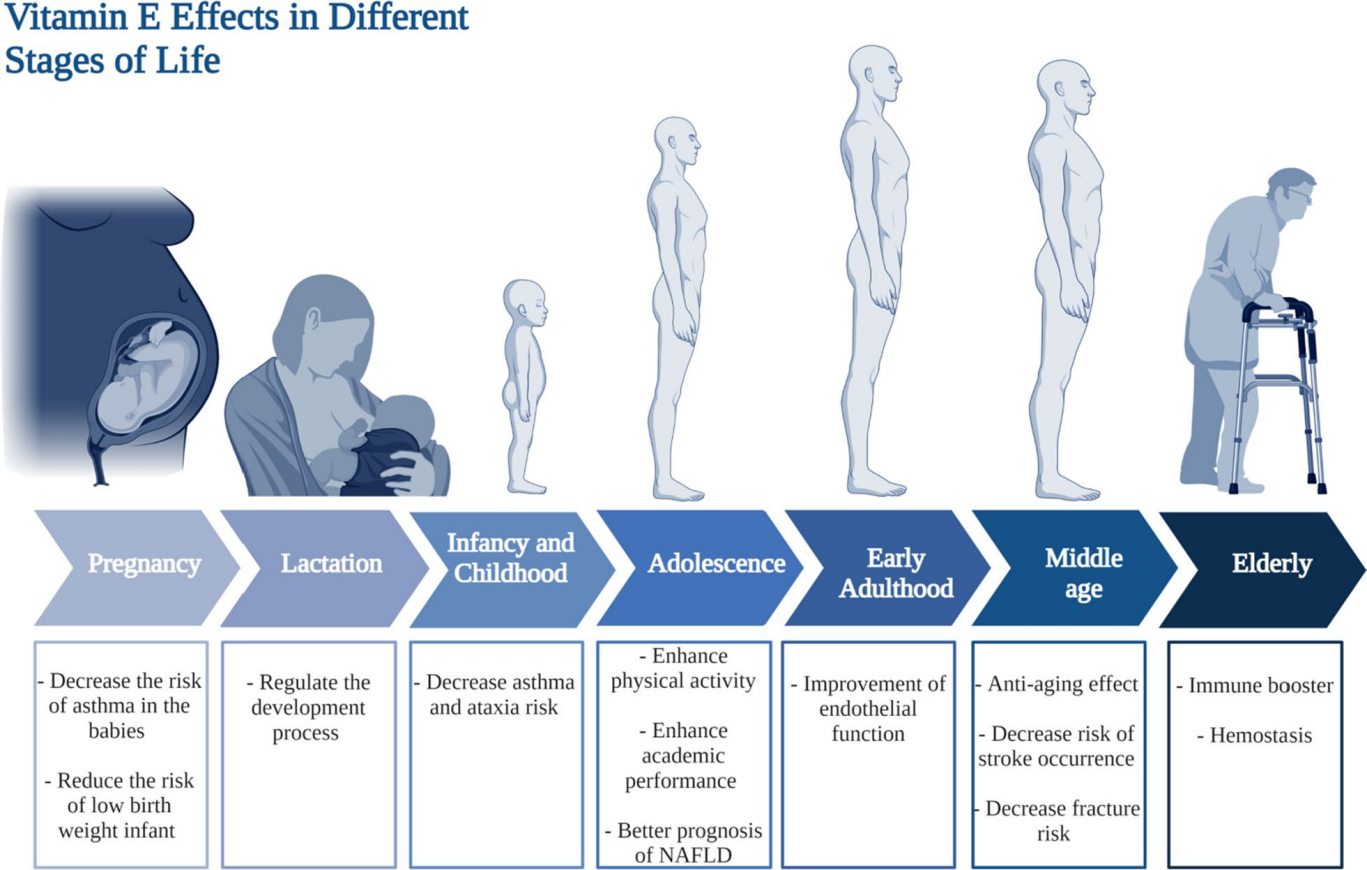
Vitamin E requirments at different stages of life. This figure represents a summary for vitamin D effects in different stages in life starting form pregnancy and lactation till the elderly population

Vitamin E influences several health outcomes during infancy and childhood. Similar to vitamin D, vitamin E plays an important role in the pathogenesis of asthma. In a clinical trial that recruited 1924 children, vitamin E intake was found to be associated with a decreased risk of children being diagnosed with asthma as shown in Fig. 5 [165]. Additionally, several reports recommend vitamin E supplementation as a potential cheap prophylactic for asthma [169]. Ataxia with vitamin E deficiency is an unusual autosomal recessive disorder [170], however all patients who suffer from ataxia since childhood should be tested for vitamin E deficiency, and consequently their treatment protocol should contain high doses of vitamin E [170]. Moreover, higher vitamin E intake during early childhood will decrease the chances of elevated levels of mid-childhood ALT [171].

#### Adolescence

Vitamin E has shown significant effect on the health of people at the adolescence age [172]. In a study done on 120 school students aged between 15 and 18 years old, high serum levels of α- and γ-tocopherol elicited an enhancement in the academic performance and students’ physical activity [172]. In addition, vitamin E could play a role in the adolescence age that leads to better prognosis in cases of non-alcoholic fatty liver disease (NAFLD) [173]. Studies have revealed that adolescent patients with NAFLD could show moderate improvement in liver biomarkers and histology when treated by tocotrienol form of vitamin E (Fig. 5) [173].

#### Adulthood

Vitamin E, the antioxidant vitamin, is essential for all age groups. A meta-analysis which included several randomized controlled trials, found a beneficial effect of vitamin E supplementation, specifically in the form of alpha-tocopherol on subclinical levels of inflammation in adults [174]. Another review that revised clinical trials involving adults 18 years of age or older, vitamin E or C supplementation alone, improve endothelial functions [175]. The importance of vitamin E supplementation for middle-aged adults cannot be over-emphasized. It serves as a protective agent against several common and serious adverse health outcomes in that age group. A randomized controlled trials evaluated the effect of a capsulated serum containing a mixture that includes vitamin E exhibiting antiaging and brightening effects of skin [176]. In terms of cardiac health, a recent meta-analysis showed a significant inverse relationship between intake of vitamin E and the risk of stroke occurrence [177]. For skeletal health, fracture risk at any site is significantly decreased when vitamin E intake is increased in men [178]. Collectively, the anti-aging and protecting effects of vitamin E are the main reasons for which vitamin E supplementation during middle age is important.

#### Elderly

Vitamin E has long been reported to act as immune booster in senior population [15, 179, 180]. Although the recommended dose for adults is 15 mg, recommended dose for elder population ranges from 400 to 800 mg daily [85]. Among 32 healthy elderlies, the group that received regular administration of 800 mg of vitamin E (α-tocopheryl acetate) has shown improvement in immunity likely mediated by decrease in plasma lipid peroxides and/or PGE2 synthesized by peripheral blood mononuclear cells (PBMCs) [179]. In parallel, recent clinical trial on elderly residents aged above 65 years old assessed the effect of vitamin E daily administration E for a year on respiratory tract infections (RTIs) susceptibility [181]. Daily use of 200 IU vitamin E per day have significantly protected subjects from upper RTIs [181]. Furthermore, risk of pneumonia was found to be remarkably lower in elderly smokers who receive vitamin E supplementation versus placebo group [182]. Additionally, vitamin E exerted haptoglobin genotype-dependent prevention of brain-related problems in type 2 diabetic elderlies [183]. Therefore, vitamin E daily supplementation would be in general highly recommended for senior population [85]. On the other hand, vitamin E deficiency happens rarely in elderlies in cases of fats malabsorption, pancreatic problems, coeliac disease, or enteritis [85]. On the other hand, hypervitaminosis E should be avoided for its accompanied complications. Daily vitamin E overdoses was linked to inhibition of platelet aggregation, diarrhea, nausea, endocrine dysfunction, cardiovascular problems, exhaustion, and muscle weakness [184].

#### Sex effect

It has been reported that vitamin E requirements among both sexes is the same which is 15 mg/day according to the national institute of health (NIH) as presented in Table 2. Yet, a recent study showed that vitamin E supplementation among males might increase the risk of developing prostate cancer [185].

## Vitamin K

Vitamin K is a well-recognized vitamin in terms of its indisputable role in hemostasis through its participation in the post-translational modifications of several coagulation factors [51]. Nevertheless, it’s also involved in connective tissue calcification [186]. Different molecular forms of vitamin K could come from different sources. For instance, vitamin K_1_ (Phylloquinone) is of plant origin. It is mostly synthesized in photosynthetic organisms such as green plants [186]. For vitamin K_2_ (Menaquinones), it is mainly synthesized by the anaerobic intestinal flora [51, 85].

### Vitamin K: metabolism, dietary sources, functions, and signaling pathways

Vitamin K that comes from diet is absorbed by micelles formation upon mixing with bile salts. The more the fatty content in the meal, the more efficient is its absorption [186]. When taken up by enterocytes, vitamin K and other dietary fats are packed together into the chylomicrons to enter the circulation [51]. While phylloquinone are being transported by the chylomicron, menaquinones is usually transported by the VLDL or low-density lipoprotein (LDL), and both are taken up the liver via lipoprotein receptors [51]. The metabolism of phylloquinone and menaquinone starts with ω-hydroxylation, then β-oxidation of the polyisoprenoic side chain into shorter carboxylic acids takes place in the mitochondria to allow glucuronidation which is followed by urine or bile excretion [187].

Vitamin K bioactive forms could be found in various sources. Menaquinones are present in bovine liver and fermented cheeses. However, menaquinones are rarely found in fish except for eel [186]. On the contrary, phylloquinones are mainly present in green leafy vegetables as previously mentioned such as lettuce, chard, parsley, spinach, cabbage, broccoli, kale, and other green cruciferous vegetables [186, 188]. As previously mentioned, vitamin K content is largely influenced by GM [10, 189, 190]. In this context, any disturbance to the GM would affect vitamin K production levels [191]. However, vitamin K could also affect GM composition [10]. A study on mice groups revealed that treatment with vitamin K could have anti-carcinogenic effect, which in turn decreases the risk of colorectal cancer via affecting GM profile [55].

Vitamin K is important for the synthesis of prothrombin and is considered as key player in posttranslational activation of coagulation factors as mentioned earlier [192]. Intriguingly, not only coagulation factors which are currently known to need vitamin-k-depended γ-carboxylation, but also Gla proteins have been found to undergo posttranslational γ-carboxylation [186]. Gla proteins have been known, so far, to be involved in blood coagulation and connective tissue mineralization [193]. Moreover, four Gla proteins function as transmembrane receptors, one binds the hyaluronic acid, one acts as growth-factor-like signaling molecule, and one is γ-carboxylase [193].

Aside of its role in prothrombin synthesis, vitamin K is also involved in the development of central nervous system (CNS) by participating in sphingolipids biosynthesis which are essential component of the brain cell membranes [53, 54]. Moreover, in the presence of nerve growth factor (NGF), vitamin K can potentiate neurite outgrowth. This action was found to be mediated by protein kinase A (PKA) and mitogen-activated protein kinase (MAPK) in neuronal PC12D cells [53, 194].

### Vitamin K profile at different life stages

#### Preconception and pregnancy

**Fig. 6:**
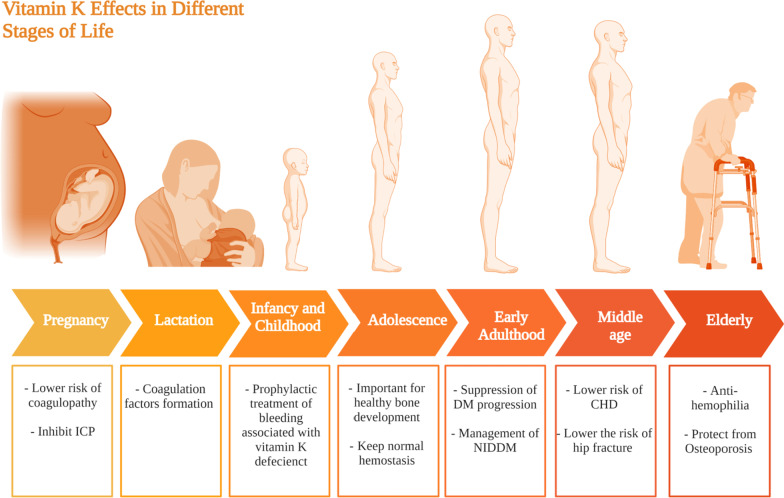
Vitamin K requirments at different life stages. This figure represents a summary for vitamin K effects at different stages in life starting form pregnancy and lactation till the elderly population

A study by Maldonado et al. reported that pregnant women who experience intrahepatic cholestasis of pregnancy (ICP): intense itching in late pregnancy due to liver condition, are considered to be at risk of vitamin K deficiency as shown in Fig. 6 and they need coagulation profile follow-up to avoid coagulopathy complications during labor [195, 196].

#### Lactation

Vitamin K is naturally present in mother’s milk [197], even though its level has been found to be low in developed countries [198]. Its supplementation to the mother increases its levels in the milk, which subsequently increases its levels in breastfed infants [197]. Nonetheless, maternal vitamin K supplementation cannot substitute vitamin K supplementation to the newly born [197]. The importance of vitamin K to the newborn, stems from its role in the formation of coagulation factors as shown in Fig. 6. Vitamin K deficiency bleeding can be life threatening [199]. It is classified as early; within the first day of birth, classic; within the first week of birth, and late; within the first 2 weeks to 6 months of age [199]. Therefore, it is of importance that all newborns receive vitamin K prophylaxis [199, 200].

#### Infancy and childhood

Similar to pregnancy, vitamin K supplementation is considered vital it serves as a prophylactic treatment of vitamin K deficiency bleeding (VKDB) [199]. VKDB usually manifests as intra-cranial or mucosal hemorrhages manifested as nodular purpura in an 9 month old infant [201]. Infants with cystic fibrosis suffer from an increased risk of developing vitamin K coagulopathy, therefore adding vitamin K supplementation to their treatment protocol would aid prevent further complications [202, 203]. On the other hand, vitamin K levels were found much higher in children with febrile seizures compared to age-matched healthy controls (33,045,823) suggesting that it has a potential role in the etiopathogenesis of febrile seizures in children. In conclusion, further studies are recommended as vitamin K supplementation’s effect on infants and children is still not that clear compared to other fat soluble vitamins.

#### Adolescence

Vitamin K is important in the adolescence age for good bone health. Between 155 and 188 μg vitamin K per day is required to maintain healthy bone development [204]. In addition, daily 54–62 μg vitamin K/Kg is needed for normal hemostasis [204]. Yet, evidences showed that during adolescence vitamin K is important for bone health more than hemostasis [204–206].

#### Adulthood

Vitamin K has several beneficial effects regarding diabetes mellitus among adults, with several studies pointing for a beneficial effect on insulin sensitivity and glycemic status of diabetic patients, leading to suppression of the progression of the disease as summarized in Fig. 6 [207, 208]. It is also worth mentioning that Vitamin K2 in particular was found to exert a better effect on type 2 diabetes mellitus patients compared to vitamin K1 [209].

#### Elderly

Previous studies have shown that vitamin K low levels has been directly associated with poor cognitive functions among senior populations [210]. Likewise, data obtained from the nationwide Cardiovascular disease in Norway suggested that a higher intake of vitamin K2 is associated with a lower risk of coronary heart disease [211]. This conclusion is supported by a study revealing that reduced levels of both vitamin D and K, is associated with adverse cardiac remodeling, and generally all-cause of mortality in men and women [212]. In terms of skeletal health, vitamin K deficiency was found to be prevalent among hip fracture patients [213].

Phylloquinone deficiency in elderly is one of the major causes of hemophilia, in particular, in the gastrointestinal tract, attributed to the absence of coagulation factors that are vitamin-K-dependent. In addition, other studies revealed the involvement of vitamin K deficiency in osteoporosis via alteration of Gla protein osteocalcin activity [92, 214]. Osteocalcin problems are usually linked to bone fractures in senior population. Nevertheless, vitamin K supplements was found to remarkably decrease the probability of hip fractures in individuals older than 75 years old [85].

#### Sex effect

It has been reported that the daily requirements of vitamin K among both sexes slightly differs as it is recommended for males to have around 120 µg/day while females requirements are fulfilled with only 90 µg/day as summarized in Table 2 [85].

## Conclusion

In conclusion, this review article is considered an updated comprehensive overview shedding the light onto the potential role of fat-soluble vitamins at every stage of human's life. During pregnancy, it has been found that vitamin A is essential for normal morphological development for the fetus while vitamin D appears to act as a protective shield against gestational diabetes and preeclampsia. Nonetheless, vitamins E was found to have essential role in decreasing the risk of developing low-birth weight infants whereas vitamin K decreases the risk of coagulopathy among pregnant women. It has been also highlighted that vitamins supplementation during lactation is of potential since some vitamins are of high prophylactic significance such as vitamin D. Vitamin D acts as a preventive measurement from autism, asthma, and rickets diseases in newborn and children. Also, it was highlighted that fat-soluble vitamins when taken in proper quantities, are critical for normal function, growth, and maintenance of tissues as highlighted, though can be toxic warranting for their administration at optimum doses. The study emphasizes on the significant difference between males and females' daily vitamins requirements to aid clinicians decide on best regimen, and as well to highlight for future needed research in that field. The crosstalk between fat-soluble vitamins and GM has also been highlighted. However, the interaction between sex tissues and fat-soluble vitamins was only outlined in case of vitamin A and D highlighting a promising gap that needs to be further studied. Our study warrants for further investigation on the roles of fat soluble vitamins in several pathological conditions such as cancer, autoimmune diseases, infectious diseases and neurodegenerative diseases.

## Data Availability

Data sharing is not applicable to this review since no datasets were generated or analyzed during the current article.
